# Fluorescence-Enhanced Immunoassay (FEIA) Platform by Combining AIE Nanobeads on a Plasmonic Device for Selective Detection of IL‑6 Cytokine

**DOI:** 10.1021/acs.analchem.5c06952

**Published:** 2026-03-04

**Authors:** Navneet Chaudhary, Xueen Jia, Nicolas Boulanger, Thomas Wagberg

**Affiliations:** † Department of Physics, 8075 Umeå University, Umeå 901 87, Sweden; ‡ Nordic Nano Biotech AB, Tvistevägen 48, Umeå 907 36, Sweden; § Wallenberg Initiative Materials Science for Sustainability, Umeå University, Umeå SE-901 87, Sweden

## Abstract

The development of advanced diagnostic systems with exceptional sensitivity and precision is important for the accurate and timely detection of biomarkers associated with inflammatory responses and autoimmune disorders. Here, we introduce a simple and high-throughput fluorescence-enhanced immunoassay (FEIA) platform for detection of cytokine interleukin (IL-6). This platform is based on a plasmonic gold substrate integrated with aggregation-induced emission (AIE) nanobeads. The plasmonic substrate is low-cost and easily scalable and can be fabricated using a straightforward plasma etching method. Our FEIA platform facilitates precise quantification of IL-6, achieving a low detection limit of 0.5 pM and a dynamic linear range from 0 to 1250 pM, manifesting the efficiency of the technique. The platform with improved fluorescence and stability of the AIE nanobeads, together with the plasmonic substrate, offers an effective tool for cytokine monitoring. By enabling easy integration into the current clinical diagnosis routine by the possible use of plate imagers and plate readers, it provides significant advancement in accuracy and diagnosis along with therapeutic outcomes for inflammatory conditions.

## Graphical abstract

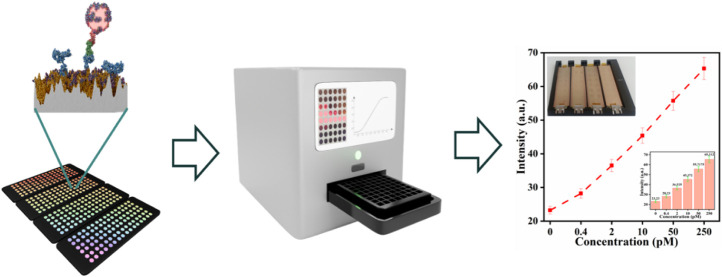

## Introduction

Inflammatory conditions and autoimmune disorders pose a major threat to global public health, requiring rapid and effective diagnostic tools to facilitate appropriate therapeutic interventions and enhance patient outcomes.^1,2^ IL-6 cytokines have evolved into a pivotal mediator in both healthy and pathological immune responses.^1,3^ It is connected to important body functions like the acute-phase response and cell signaling, but when it does not work properly, it can lead to severe health problems, including autoimmune diseases, various cancers, liver diseases, and long-term inflammation issues.^4,5^ Elevated IL-6 levels are commonly detected in patients with serious illnesses and have been recognized as accurate biomarkers for determining disease progression and therapeutic response.^6^ Hence, being able to detect small amounts of IL-6 more accurately is very important for early diagnosis and ongoing patient monitoring, pushing the development of new biosensing technologies. Traditional methods for monitoring IL-6, like lateral flow immunoassay (LFA),^7^ radioimmunoassay (RIA), quantitative PCR, flow cytometry, and Western blotting,^8–11^ are still often used in research and clinical laboratories. However, these approaches have some disadvantages, as they typically require high analyte concentrations to produce consistent results, are comparatively low in sensitivity, and are generally accompanied by lengthy processes.^12^ Furthermore, traditional methods are typically inadequate for real-time or point-of-care applications. A need for faster, more sensitive, and reliable detection systems is therefore motivated.^13^ Recent advancement in nanotechnology have transformed biomolecular detection at the core, and now provides exceptional possibilities for evolving next-generation biosensors.^14^ Among these, plasmonic substrates using gold nanoparticles have attracted significant interest due to their unique optical characteristics, good stability, inertness, biocompatibility, and ease of functionalization.^13,15^

The localized surface plasmon resonance (LSPR) effect of gold significantly amplifies local electromagnetic fields, leading to significant enhancements in fluorescence signals from nearby fluorophores.^16^ This feature has been used to make biosensing methods more sensitive and specific for various biomolecules, including cytokines like IL-6. Using fluorescent aggregation-induced emission (AIE) nanobeads combined with gold surfaces significantly enhances the efficacy of plasmonic biosensors.^17^ AIE-based nanobeads have exceptional intensity, photostability, and resistance to photobleaching, enabling the detection of cytokines at concentrations far lower than those detectable by conventional techniques.^18^ When combined with a plasmonic substrate at an optimal separation distance of approximately 15–20 nm, these AIE nanobeads exhibit enhanced signal amplification due to well-established plasmon–fluorophore energy transfer mechanisms, thereby improving the sensitivity and reliability of the developed detection approach. The synergistic combination of the plasmonic substrate and AIE nanobeads establishes a robust detection system that facilitates real-time monitoring, essential for clinical decision-making and point-of-care diagnosis.^17,19–21^

Here, we introduce an innovative approach for detecting IL-6 utilizing AIE nanobeads on a nanostructured plasmonic substrate, addressing the limitations of traditional methods; see Figure 1. The capability to determine low amounts of IL-6 in real-time facilitates early diagnosis and ongoing clinical surveillance of inflammatory and autoimmune disorders.

**Figure 1:**
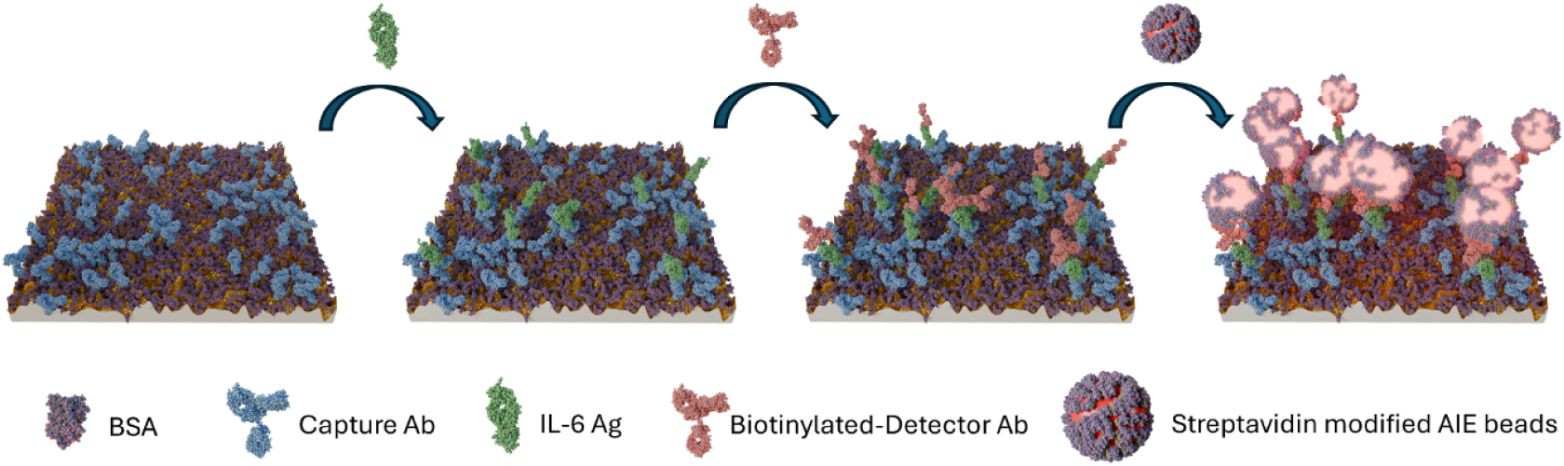
Schematic illustration of FEIA for IL-6 detection on a functionalized plasmonic substrate with AIE nanobeads.

## Experimental Section

### Reagents and Chemicals

IL-6 protein and antibody were purchased from Sino Biological Inc., USA; poly­(tetra phthalate) (PET) and nanofluorophore nanobeads (NARPC) were provided by AIE Institute in Guangzhou, China; 11-Mercaptoundecanoic acid (MUA), 1-ethyl-3-(3-dimethylaminopropyl)­carbodiimide (EDC), and *N*-hydroxysuccinimide (NHS) were purchased from Sigma-Aldrich; and fetal bovine serum albumin (BSA) was purchased from Fisher Chemicals. Unmodified streptavidin was purchased from Sigma-Aldrich. Biotin, phosphate-buffered saline, and tween 20 were purchased from Thermo Fisher Scientific. The physical vapor deposition (PVD) used during evaporation was bought from Kurt J. Lesker and included tungsten boats and 99.99% pure gold pellets. RS France made the microscope glass used, and the atomic force microscopy (AFM) cantilever (spring constant k = 0.5 N) was bought from Mikromash Europe.

### Fabrication of Plasmonic Substrate

#### Surface Modification by Plasma Etching

PET was investigated as a potential plasmonic substrate in this study. To enhance its surface characteristics, a systematic optimization of plasma etching parameters and other key variables was conducted. The optimization process involved varying key factors such as the process gases, specifically oxygen and argon-exposure time, and radio frequency (RF) power. In the final experiment design, the RF power was fixed at 200 W, while the etching time was varied at 0, 10, 20, and 30 min. Based on the optimization, oxygen gas was selected as a plasma source, with an RF power of 200 W and an optimized etching time of 30 min.

To evaluate the surface modification, AFM (Park NX-Hivac, Korea) was employed after each plasma treatment and a subsequent gold coating for 30 min. Detailed plasma treatment data are provided in the Supporting Information (Figure S1). As shown in Figure 2a (2D image) and 2c (3D image), the surface topography of the untreated PET substrate (0 min) appears smooth with negligible roughness. In contrast, Figure 2b and d, depicting the PET substrate after 30 min of plasma etching, exhibit significant surface roughness and surface texture. A quantitative comparison presented in Figure 2e reveals that the untreated PET substrate exhibits a low roughness of approximately 7.5 nm, whereas the 30 min plasma-treated substrate demonstrates a significantly increased roughness of up to 37 nm, which increases the effective surface area and improves interfacial interactions between the gold film and the substrate. This improved topography led to better film anchoring and thus enhances the long-term stability of the PET substrate.

**Figure 2:**
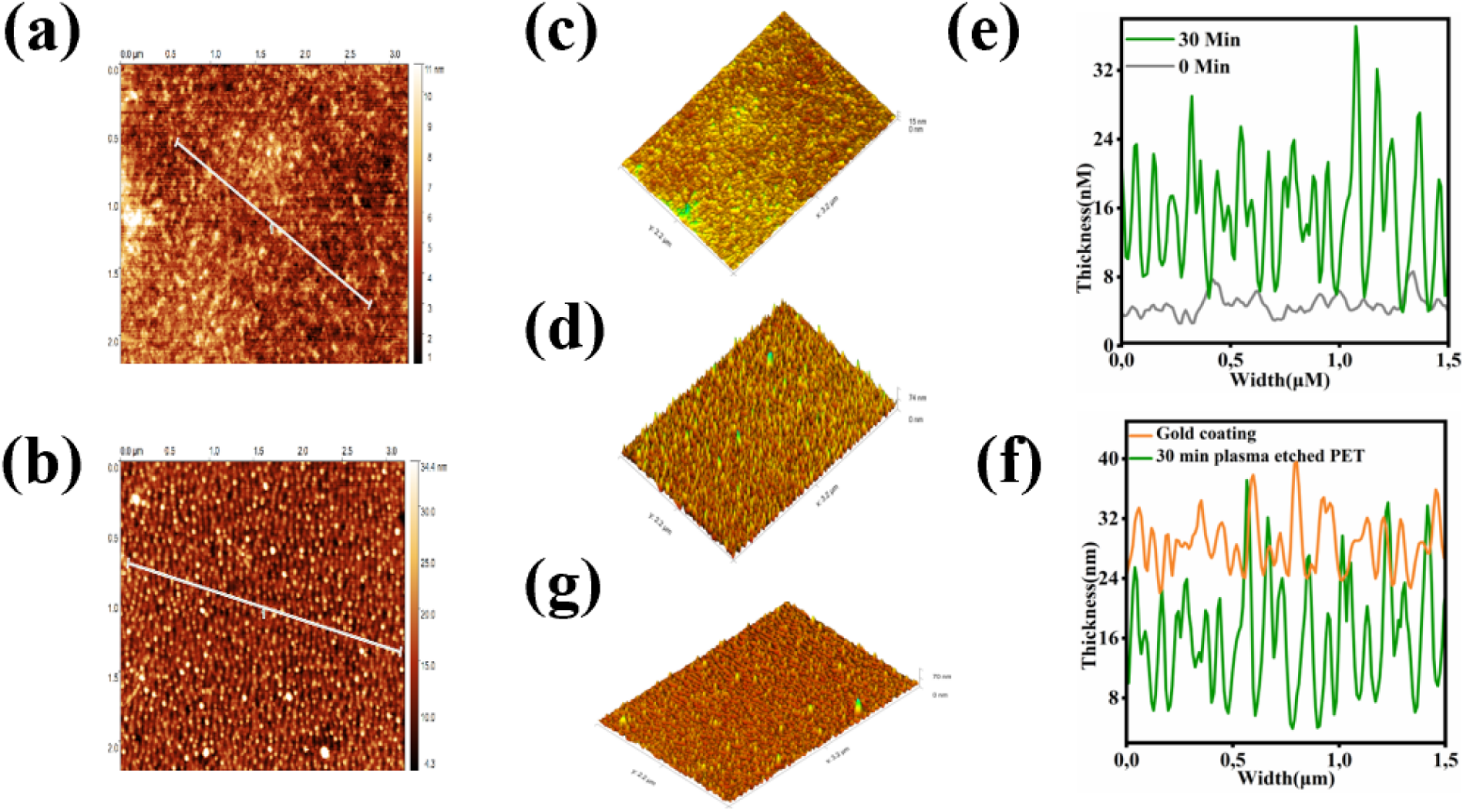
Surface modification and gold coating on PET substrates: (a) 2D AFM topography before plasma etching; (b) 2D AFM topography after plasma etching; (c) 3D AFM surface profile before etching; (d) 3D AFM surface profile after etching. (e) Presentation of a comparison of surface roughness between the untreated (gray) and 30 min plasma-treated (green) substrates. (f) Displays the cross-sectional profile of the substrate after deposition of a 30 nm gold thin film (gold color), compared to the cross-sectional profile of the 30 min plasma-treated substrate (green color). (g) Provides a 3D visualization of the gold-coated PET substrate.

#### Gold Deposition after Plasma Etching via PVD

A PVD system (Kurt J. Lesker Co.) was employed to form gold thin films onto the PET substrates via physical vapor deposition. Gold pellets were heated in a tungsten boat under high vacuum until vaporization, enabling gold atoms to uniformly deposit on the PET substrate. The deposition rate and film thickness were constantly tracked and regulated using a quartz crystal sensor, which adjusted the supplied power to ensure uniform film formation. In the next phase of the study, the final thickness of the gold layer was controlled so that the thickness was maintained between 28 and 30 nm. We also evaluated several gold film thicknesses from 0 to 30 nm deposited on PET substrates to identify the most suitable surface for antibody immobilization and plasmon-enhanced fluorescence. After plasma activation and subsequent characterization, the 30 nm gold layer showed the most favorable surface properties. This thickness provided an optimal nanoscale roughness that improved antibody binding efficiency and created a more uniform functional surface. In addition, the 30 nm film supported stronger plasmon–light interactions compared to thinner layers, enabling more efficient excitation of fluorophore signals. Thicker films (>30 nm) resulted in reduced plasmonic activity and poorer surface activation. Therefore, 30 nm gold was selected as it provided the best balance between surface chemistry for robust biofunctionalization and optical performance for enhanced fluorescence detection. Supplementary Figure S2 shows the dependence of the gold film thickness on the deposition time, highlighting the changes observed between 0 and 30 min. Figure 2f illustrates the improved interfacial contact between the gold thin film and the 30 min plasma-treated PET substrate. The lower graph (green) shows the roughness of the plasma-etched surface ranging between 4 and 34 nm, while after gold coating, the roughness (orange) varies approximately from 24 to 40 nm. This supports the explanation in Figure 2e, demonstrating how the 30 min treated surface helps anchor the gold particles onto the rough PET substrate, resulting in a uniform film. The 3D surface morphology shown in Figure 2g reveals distinct gold clusters that are well anchored to the modified substrate, highlighting the effectiveness of plasma etching in preparing PET for stable gold film deposition.

#### Functionalization of Plasmonic Substrate

Freshly gold-coated plasmonic substrate was immersed overnight at room temperature in a solution of 10 mM MUA prepared in ethanol (1:3). MUA was applied to modify the plasmonic surface morphology due to its thiol (−SH)-containing group, which exhibits strong affinity toward gold, enabling the formation of a self-assembled monolayer. The substrate was thoroughly rinsed and dried. Surface functionalization was then carried out using a well-known EDC/NHS activation protocol. Equal amounts of 20 mM EDC and NHS were freshly prepared and incubated with the MUA-modified plasmonic surfaces for 30 min. In this process, EDC activates the carboxyl (−COOH) groups of antibodies, forming a reactive O-acylisourea intermediate. However, due to the instability of this intermediate in aqueous environments, NHS is introduced to convert it into a more stable NHS-ester. This NHS-activated ester readily reacts with the primary amine (−NH_2_) group on the capture antibody, forming a very stable covalent amide bond between the antibody and the gold-coated surface. This strategy ensures efficient, oriented, and stable immobilization of the capture antibody on the fabricated plasmonic substrate.^22–24^

### Labeling and Optical Characterization of Labeled AIE Nanobeads (NARPC) with Streptavidin

Aggregation-Induced Emission (AIE) nanobeads were used in this study because they provide significant advantages over conventional fluorescent nanoparticles for immunoassay applications. These beads are composed of polystyrene spheres functionalized with surface −COOH groups, enabling efficient and stable bioconjugation with detection antibodies. The fluorescent component embedded in these beads exhibits AIE behavior, meaning the fluorescence intensity increases upon aggregation instead of suffering from aggregation-caused quenching (ACQ), which is a common limitation of traditional organic dyes and quantum dots. Because AIE fluorophores become brighter when immobilized or clustered—conditions that naturally occur during antibody–antigen complex formation—they are particularly well-suited for signal amplification in solid-phase immunoassays. This property enhances assay sensitivity, improves the signal-to-noise ratio, and reduces quenching effects that typically occur on solid substrates or at high local dye density.

AIE nanobeads were labeled with streptavidin using a standard EDC/NHS coupling protocol as described in substrate functionalization section. Initially, 100 μL of AIE nanobead solution (1 wt %) was diluted in 500 μL of MES buffer (pH 6.0), washed once by centrifugation, and the supernatant was discarded. The nanobeads were then redispersed in 500 μL of fresh MES buffer, followed by the addition of EDC and NHS at the final concentration of 3 mg/mL in a 1:3 molar ratio. The suspension was gently rotated in the dark for 30 min to activate the carboxyl group on the nanobeads’ surface. After activation, the beads were washed again to remove excess reagent. Next, the nanobeads were redispersed in 500 μL of MES buffer (pH 6.5) and incubated with 40 μg of streptavidin under gentle rotation and shaking in the dark for 2 h. The solution was centrifuged, and the supernatant was removed. To minimize the nonspecific binding, the beads were treated with 500 μL of blocking solution (1% glycine and 0.05% BSA) and incubated under the same conditions for 1 h, followed by a washing step. Finally, the nanobeads were incubated in 500 μL of preservation solution (Tris 20 mM, 0% tween 20, and 0.5% BSA), washed once more, and labeled nanobeads were resuspended in 100 μL of preservation buffer for storage at 4 °C until further use.

The optical characterization of the streptavidin-labeled AIE nanobeads was performed using a fluorescence spectrophotometer (Edinburgh Instruments FLS1000). Figure 3a shows the EDC/NHS-mediated conjugation of NARPC to streptavidin. In this process, EDC activates the carboxyl groups on the NARPC nanobeads, and NHS stabilizes them by forming reactive NHS esters. These esters then react with the primary amines on streptavidin, creating stable covalent amide bonds and effectively linking NARPC to streptavidin. Figure 3b illustrates the overall labeling representation of NARPC nanobeads with streptavidin. Figure 3c shows the microscopic image of the unlabeled AIE nanobeads, with an average diameter of approximately 200 nm. Figure 3d and e represents the excitation and emission spectra of the nanobeads before and after streptavidin labeling, respectively. In Figure 3d, three distinct excitation peaks were observed at 318, 360, and 475 nm; red and blue spectra represent nanobeads prior to and post labeling, respectively. The excitation peak positions and intensities remained consistent after labeling, indicating that the optical properties of the nanobeads were unchanged. These multiple excitation wavelengths offer flexibility in assay design. In this study, 365 and 475 nm excitation wavelengths were employed in two FEIA platforms: one integrated with a glass slide using a plate imager at a 475 nm excitation wavelength and another based on a 96-well microtiter plate format using a plate reader at a 365 nm wavelength. Figure 3e displays the emission spectra of the labeled nanobeads excited at 470 nm. The emission peak at 605 nm remained the same after labeling, confirming that the fluorescence characteristics were retained as excitation spectra. The inset in Figure 3e shows the visual comparison of the labeled nanobeads under normal daylight and UV light, further supporting the successful labeling of streptavidin while maintaining the fluorescence of the nanobeads. For additional information, Supplementary Figure S3 shows a microscopic image of AIE beads after labeling with streptavidin.

**Figure 3:**
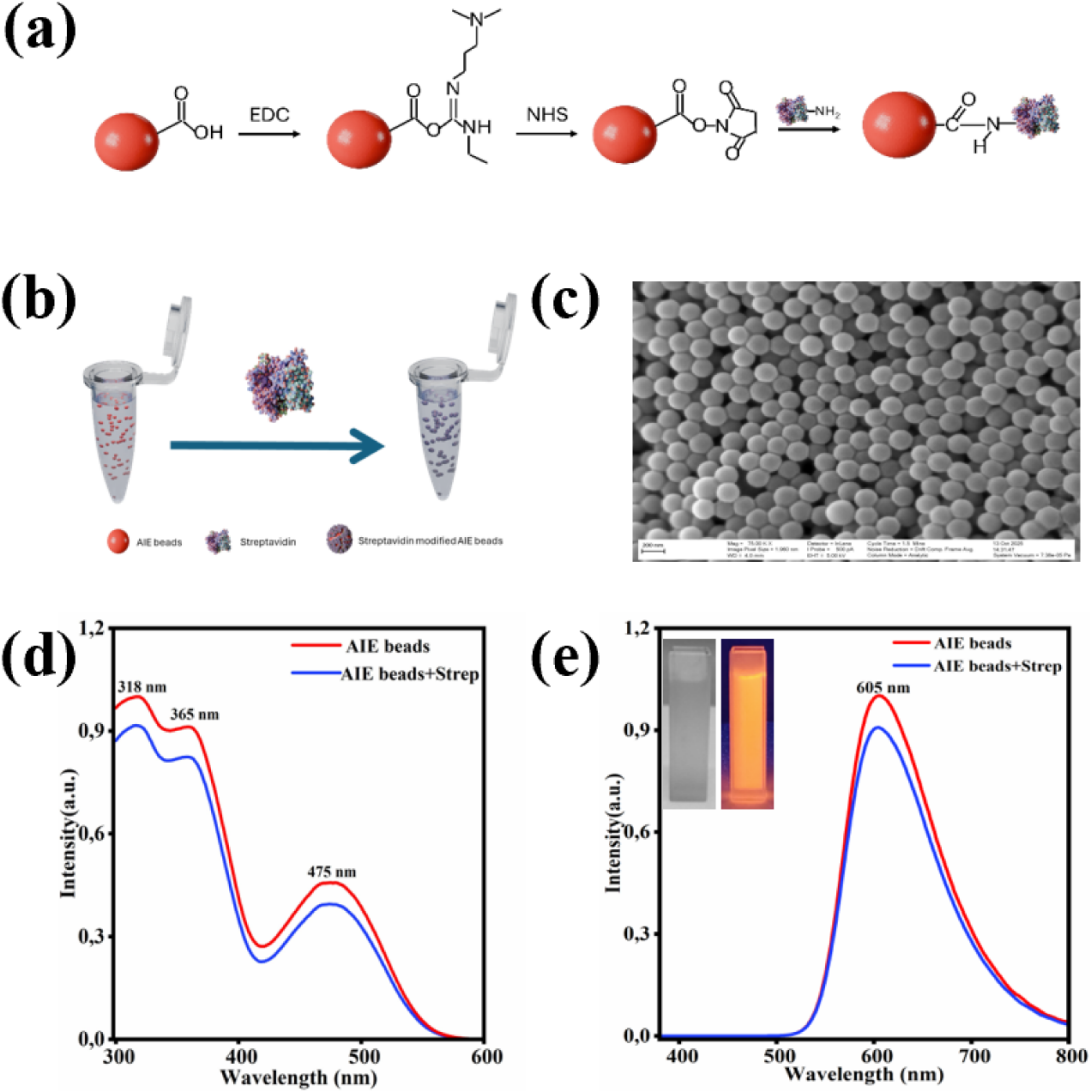
Illustration of the labeling process of NARPC: (a) EDC/NHS-mediated conjugation of NARPC to streptavidin; (b) schematic representation of the labeling process; (c) SEM image of unlabeled NARPC; (d) excitation spectrum of NARPC before and after labeling; and (e) emission spectrum of NARPC before and after labeling.

### Preparation of Biotin-Labeled Detection Antibody

Biotinylating of secondary antibodies is a prevalent technique employed in immunoassays to enhance signal detection with streptavidin- or avidin-based systems. This procedure typically employs *N*-hydroxysuccinimide (NHS)-activated biotin, which adheres to the lysine residues of the antibody. The biotinylating reaction involves combining NHS-biotin in dimethyl sulfoxide (DMSO) with the antibody, utilizing 20–70 μg of NHS-biotin per mg of antibody, and allowing the mixture to incubate for 30–120 min at room temperature. Following the reaction, unreacted biotin is effectively eliminated using dialysis to avert nonspecific binding.^25,26^

### Integration of Plasmonic Substrates onto Glass Slides and 96-Well Microtiter Plates

In this study, we established two different setups for integrating plasmonic substrates into immunoassay platforms. In the first approach, the plasmonic substrate was assembled on a glass slide, and wells were created using a plate sealer, allowing the entire procedure to be conducted within these wells; see Figure 4a. This method is simple, straightforward, and time-efficient. In the second approach, the plasmonic gold substrate was placed under a 96-well microtiter plate, allowing it to be used like a regular ELISA plate, as shown in Figure 4c.

**Figure 4:**
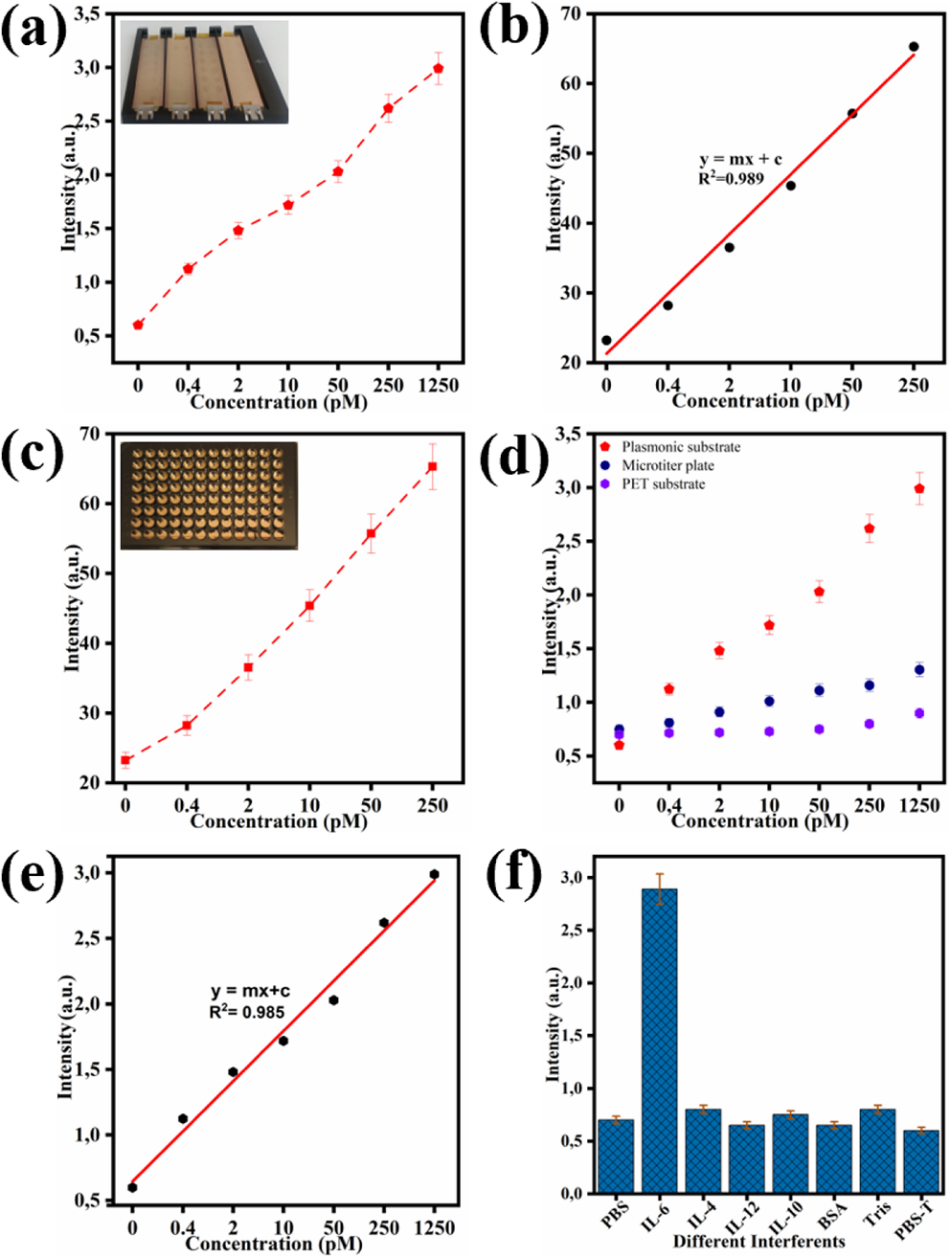
FEIA performance on different platforms: (a) sensing response for IL-6 ranging from 0 to 250 pM on the glass slide-based setup; (b) corresponding linear calibration curve; (c) sensing response for IL-6 ranging from 0 to 1250 pM on the 96-well microtiter plate; (d) comparison of fluorescence intensity between the gold-coated microtiter plate, uncoated plate, and uncoated PET substrate; (e) linear calibration curve obtained from the microtiter plate assay; (f) interference study showing specificity toward IL-6 over other cytokines.

## Results and Discussion

### Immunoassay Conducted on Plasmonic Substrates Assembled on Glass Slides

In this setup, the FEIA platform was developed onto a glass slide, as detailed in plasmonic substrate integration section. Monoclonal capture antibodies specific to the IL-6 cytokine (2 μg/mL) were prepared in 1× PBS buffer and immobilized onto the plasmonic slide in a 6 × 2 spot matrix. A volume of 6 μL was applied to each spot, followed by overnight incubation at 4 °C. The next day, the plasmonic slides were washed four times with P-BST (PBS containing 0.5% Tween 20) to remove unbound antibodies. Subsequently, nonspecific binding sites were blocked by incubating the plasmonic slide with 2% BSA for 1 h at room temperature (RT). Following blocking, IL-6 antigen was added at varying concentrations (0 to 250 pM) and incubated for 2 h at RT with gentle shaking. After the incubation, the plasmonic slide was washed four times with P-BST to remove unbound antigen. A biotin-conjugated detection antibody against IL-6 (0.5 μg/mL) was then applied and incubated for 1 h at RT with gentle shaking. Finally, streptavidin-labeled AIE nanobeads were introduced as the final layer of the immunoassay and incubated for 30 min, where they served as high-brightness fluorescent reporters. Owing to their strong photostability, resistance to self-quenching, and excellent compatibility with bioconjugation chemistry, these nanobeads are particularly well-suited for amplified plasmon-enhanced fluorescence detection. Consequently, the incorporation of AIE nanobeads directly contributes to an increased fluorescence output and enables an overall superior analytical performance of the FEIA system. The plasmonic slides were finally washed as previously described, and fluorescence signals were recorded using a plate imager at an excitation wavelength of 475 nm.

The IL-6 concentration-dependent response was observed over a range from 0 to 250 pM. The resulting FEIA platform exhibits a very strong fluorescence signal that increased proportionally with increasing concentration of IL-6, and Figure 4a manifests a high sensitivity and specificity of the developed FEIA platform. Figure 4b represents a linear calibration plot corresponding to the same concentration range, showing excellent linearity with an R^2^ value of 0.989. The calculated limit of detection (LOD) was 0.52 pM based on the linear regression equation (Y = mx + c) by using the formula LOD = 3 × σ/m, where σ is the standard deviation of the intercept, m is the slope of the calibration curve, and 3 is a constant value.

To further characterize the stepwise assembly of the immunoassay, surface morphology was analyzed using AFM and SEM. This analysis provided visual confirmation of surface modifications after protein immobilization and nanobeads attachment. Corresponding images are provided in the Supporting Information (Figure S4).

To further confirm the high performance of plasmonic devices, we have compared the fluorescence signals of AIE beads on our homemade plasmonic substrate and a commercial plasmonic substrate. The results are shown in Supplementary Figure S5: AIE beads on both plasmonic substrates gave strong fluorescent signal, but there is almost no signal on untreated PET under the same concentration. We note that our plasmonic substrate is easy to make and very cheap compared to the commercial one. Additionally, the performance of the developed setup was evaluated using spiked human serum samples, and the corresponding results are presented in the Supporting Information (Figure S6).

### Immunoassay Conducted on Plasmonic Substrate Assembled on 96-Well Microtiter Plate

The FEIA platform was further evaluated by integration into a 96-well microtiter plate setup. Additionally, comparisons were made with a standard microtiter plate and plasma-treated PET substrate. The fluorescence responses obtained from the plasmonic plate were highly consistent with those from the glass slide-based FEIA platform. These findings demonstrate that the glass slide setup is capable of detecting IL-6 concentration with similar efficiency to the conventional 96-well plate format. Moreover, the glass slide-based setup offers additional advantages, such as requiring smaller volumes of critical reagents, including antibodies and antigens, hence improving cost-efficiency and reducing sample consumption.

As shown in Figure 4c the developed platform exhibits a strong fluorescence response across a broad linear concentration range of IL-6 (0 to 1250 pM). Figure 4d highlights the difference in sensing performance among the prepared plasmonic substrate, the standard microtiter plate, and the untreated PET substrate. The highest fluorescence signal is achieved only with the prepared plasmonic substrate, whereas both the standard microtiter plate and untreated PET produce low signals. The plasmonic substrate demonstrated a markedly increased fluorescence intensity of 2.99, in contrast to 1.30 for the normal microtiter plate and 0.90 for the untreated PET substrate. This is a 2.3-fold (130%) improvement compared to the microtiter plate and a 3.3-fold (232%) enhancement in relation to the untreated PET substrate. Moreover, the enhanced signal resulted in an improved limit of detection (LOD) and increased analytical sensitivity, which can be enhanced up to 500-fold in comparison with the plasmonic device and PET only. Additionally, when a similar experiment was performed using a commercially available IL-6 detection kit, the results in Supplementary Figure S7 suggest that our method exhibited superior sensitivity, with a lower detection limit of 0.5 pM compared to approximately 4 pM for the commercial kit. The linear calibration curve shown in Figure 4e demonstrates high correlation (R^2^ > 0.985), confirming the assay’s robust quantitative capability. The LOD was calculated to be 0.6 pM, as described in plasmonic slides immunoassay section. Furthermore, specificity evaluation presented in Figure 4f confirms the high selectivity of the developed platform toward IL-6, with negligible cross-reactivity against other cytokines such as IL-4, IL-8, IL-10, and IL-12. These results highlight the FEIA platforms’ strong potential for the sensitive and specific detection of IL-6 only.

### Evaluation of 96-Well Plate-Assembled Plasmonic Substrate-Based Immunoassay in Spiked Blood Serum

Based on our prior findings with PBS buffer, we extended the study using the application of the 96-well plate assembled with a plasmonic substrate-based FEIA platform for real biological samples. Figure 5a displays the fluorescence response curves obtained from spiked human serum samples over a concentration range of 0 to 1250 pM (same as PBS buffer). Figure 5b shows a comparison of the fluorescence intensity in phosphate-buffered saline (PBS) and spiked blood serum, clearly showing that the developed FEIA platform keeps a strong signal and sensitivity in the blood serum sample.

**Figure 5:**
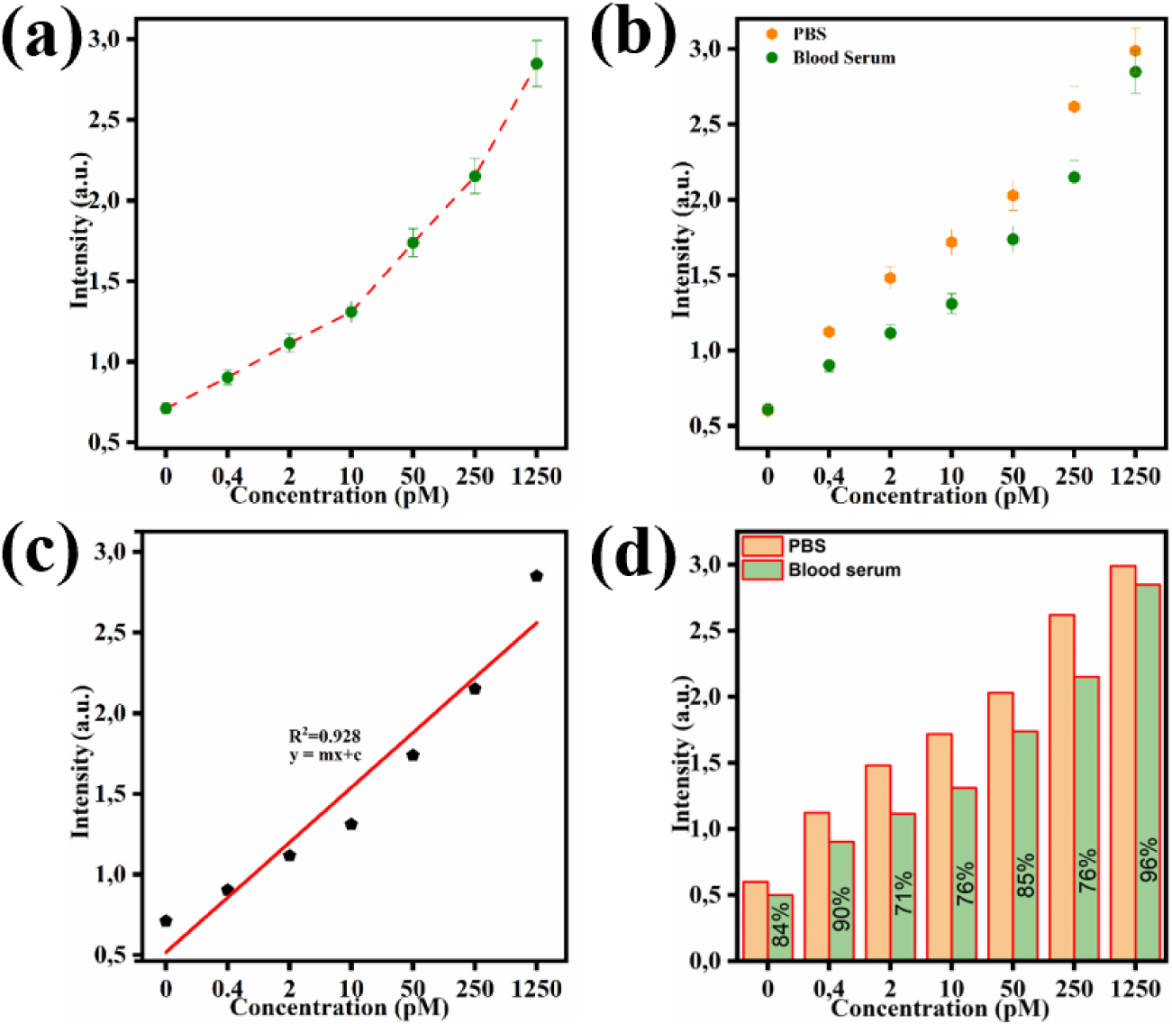
FEIA performance in spiked blood serum: (a) sensing response for IL-6 in the range of 0 to 1250 pM; (b) comparison of IL-6 detection in PBS and spiked blood serum; (c) linear calibration plot for IL-6 detection in blood serum; (d) recovery analysis plot demonstrating the assay’s accuracy.

Figure 5c presents the calibration curve, indicating linearity (R^2^ > 0.928), close to PBS. Figure 5d depicts the recovery analysis in blood serum, revealing recovery percentages ranging from 71% to 96%, hence confirming the platform’s reliability and accuracy of the developed FEIA platform in spiked blood serum. Briefly, recovery was calculated by comparing the fluorescence intensity of IL-6 standards prepared in PBS (reference matrix) with those spiked into diluted human serum (test matrix). The recovery (%) for each concentration was determined using the following equation:

1 $$\mathrm{Recovery}\left(\%\right)=\left(\frac{I_{\mathrm{serum}}}{I_{\mathrm{PBS}}}\right)\times 100$$

where

$$I_{\mathrm{PBS}}=\mathrm{fluorescence intensity of the analyte in PBS}$$

$$I_{\mathrm{serum}}=\mathrm{fluorescence intensity of the same analyte concentration spiked in serum}$$

This calculation quantifies how much signal is retained in the serum matrix relative to PBS. The data collectively demonstrate the platform’s reliability, specificity, and practical applicability for detecting IL-6 in real samples.

In Table 1 our detection platform is compared to earlier reports of IL-6 detection via various analytical techniques. As seen, the FEIA platform exhibits higher sensitivity, a lower limit of detection (LOD), and a broader linear detection range in comparison with reported immunoassays.

**Table 1:** Comparison of the Performances of Methods for the Detection of IL-6

Detection Technique	Platform/Principle	LOD (pM)	Dynamic Range (pM)	Ref.
Colorimetric/solution-base	Aggregation of AuNPs conjugated with two complementary “sandwich-type” aptamers and color change from red to pink	67,100 pM	139,240–5,274,684 pM	^27^
Colorimetric/LFIA	Anti-IL-6 antibodies conjugated to AuNPs on conjugate pad, anti-IL6 and antimouse antibodies on T and C lines, respectively	16.03 pM	52.74–379,747 pM	^28^
Colorimetric/LFIA	Application of selenium nanoparticles as labels for conjugation with anti-IL-6 detection antibody	4.22 pM		^29^
Fluorescence/fiber optic	Coating of streptavidin-modified fiber surface with biotinylated capture antibodies and conjugation of detection antibodies with three different fluorescent magnetic beads	0.60 pM	0.60–9.5 pM	^30^
Fluorescence/LFIA	Application of CdSe QDs as label for conjugation with detection antibody and utilization of two image processing software for quantification of results	1380 and 2280 pM	0–20,000 pM	^31^
SERS/label free	Aptamer-functionalized AuNPs array based on analyzing the change in the I660/I736 ratio of the SERS intensity derived from the guanine and adenine bases in the aptamer sequence	0.8 pM	10–12 pM	^32^
Colorimetric Elisa	Sandwich	6.76 pM	3–190 pM	^33^
**Current work**	**FEIA**	**0.52 pM**	**0–1250 pM**	**This work**

The enhanced sensitivity of the FEIA platform arises from the strong plasmonic amplification provided by the gold-coated substrate in combination with bright, nonquenching AIE-based fluorescent nanobeads. The plasmonic surface generates an intense localized electromagnetic field that enhances both the excitation and emission of the fluorophores, while the AIE nanobeads deliver stable, high-intensity fluorescence without self-quenching. This synergistic plasmon–fluorophore coupling significantly improves the signal-to-noise ratio, enabling selective and ultrasensitive detection of IL-6. Consequently, effective signal amplification is achieved even at low analyte concentrations, leading to an improved detection limit.

## Conclusion

We have developed a new FEIA platform for sensitive detection of IL-6 cytokines on a novel plasmonic device by using unique AIE nanobeads as a fluorescent reporter. The fluorescent immunoassay signal was significantly enhanced when AIE beads and plasmonic gold clumped together. By using this new FEIA platform, we were able to detect IL-6 at much lower levels than those tested by conventional methods such as ELISA. This novel method shows high sensitivity and specificity while facilitating real-time monitoring, fulfilling a critical need in the early identification and management of inflammatory and autoimmune disorders. The substantial use of AIE nanobead-based fluorescent immunosensors for IL-6 detection illustrates their huge potential to improve cytokine monitoring, aiding the advancement of enhanced diagnostic tools and therapeutic techniques in healthcare. Subsequent research will concentrate on enhancing this platform for wider applications in biomarker identification and point-of-care diagnostics.

## Supplementary Material



## Notes

Additional characterization data of plasmonic substrate and AIE beads (AFM and SEM, etc.); comparison of the detection performance of FEIA platform and commercial ELISA kit (PDF)
