# Supplementary material for: Fluorescence-Enhanced Immunoassay (FEIA) Platform by Combining AIE Nanobeads on a Plasmonic Device for Selective Detection of IL‑6 Cytokine
Source: Anal Chem. 2026 Mar 4;98(10):7462–70. doi: 10.1021/acs.analchem.5c06952 (PMC13000875; doi:10.1021/acs.analchem.5c06952)
Supplement: Supplementary file 1 [file ac5c06952_si_001.pdf]

Supporting information

Fluorescence-Enhanced Immunoassay (FEIA) Platform by Combining AIE Nanobeads on a Plasmonic Device for Selective detection of IL-6 Cytokine

Navneet Chaudhary[1], Xueen Jia[1,3]*, Nicolas Boulanger[1] and Thomas Wagberg[1,2]*

[1] Department of Physics, Umea University, Umea, Sweden

[2] Wallenberg Initiative Materials Science for Sustainability, Umeå University, Umeå SE-901 87, Sweden.

[3] Nordic Nano Biotech AB, Tvistevägen 48, 907 36, Umeå, Sweden

*Corresponding Authors

Email: thomas.wagberg@umu.se(Thomas Wågberg)

Email: xueen.jia@umu.se (Xueen Jia)

Table of content

S1: AFM characterization of PET substrate by plasma etching

S2: The image of size-controllable plasmonic substrate after gold coating by PVD

S3: SEM image of AIE beads after labelling with streptavidin

S4: AFM and SEM images of functionalized plasmonic substrates before and after incorporation of AIE nanobeads in the immunoassay system

S5: Comparison of nanobead performance on the developed plasmonic nanochip

S6: FEIA performance in spiked blood serum on glass slide setup.

S7: ELISA calibration and detection results for IL-6 using a commercial assay kit.

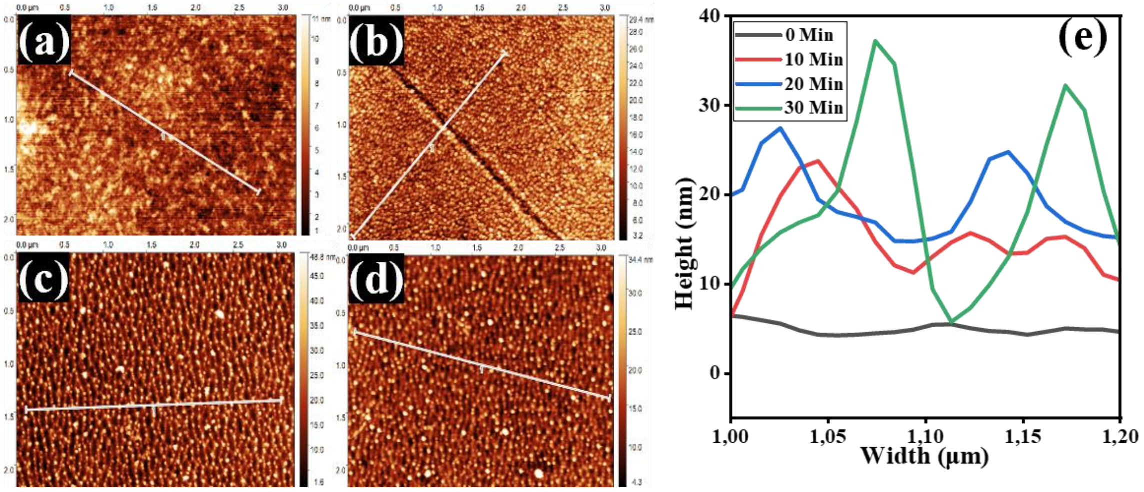

Figure S1: Plasma etching progression on PET substrates over time: (a) surface morphology of the untreated PET substrate (0 min plasma exposure); (b–d) surface modifications observed after plasma treatments of 10, 20, and 30 minutes, respectively; (e) corresponding thickness profile of plasma expo sure time.

In figure S1(e) the valley observed in the green curve at intermediate etching time reflects a temporary and localized surface reorganization during plasma treatment. At this stage, certain regions of the substrate undergo partial smoothing or reduced etch rates, which results in thickness values approaching those of the unetched (0 min) surface and causes the profile to momentarily touch the zero-reference level. However, with extended etching up to 30 minutes, the plasma treatment becomes more effective, producing a significant increase in surface roughness and thickness variation. This enhanced roughness is beneficial for gold deposition, as it improves film adhesion and promotes stronger plasmonic enhancement in the final device.

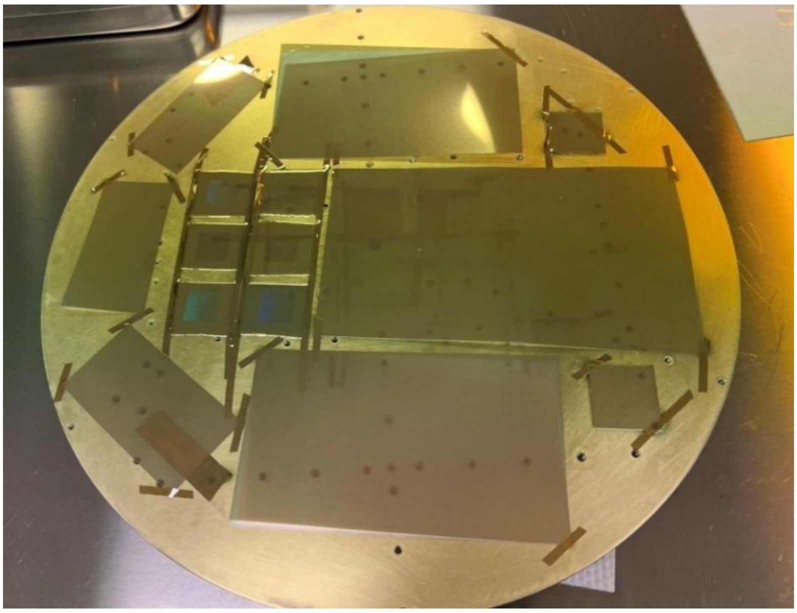

Figure S2: The image of size-controllable plasmonic substrate after gold coating by PVD.

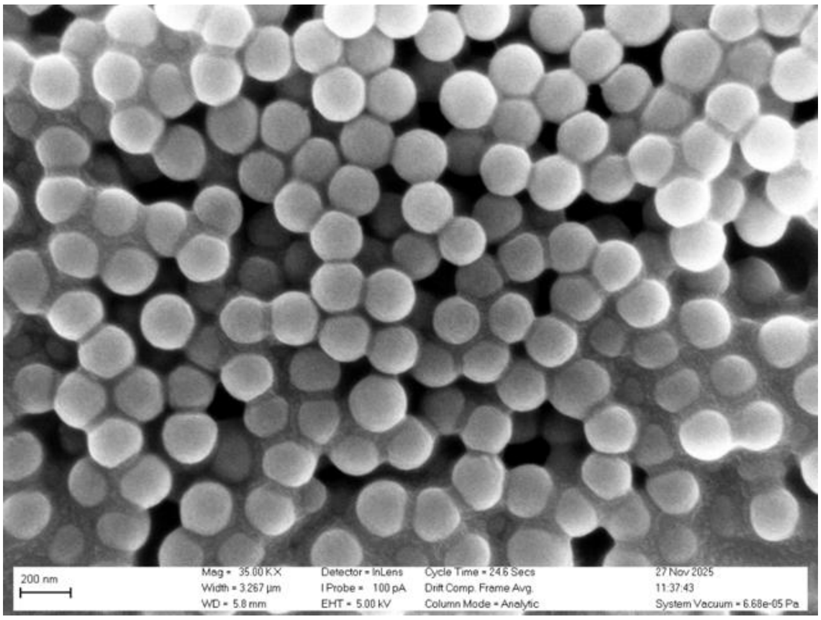

Figure S3: SEM image of AIE beads after labelling with streptavidin

Figure S3 shows the spiked serum analysis performed using the FEIA platform on the glass slide–based setup demonstrates that this configuration can detect low concentrations of IL-6 in spiked blood serum samples. The observed sensing response confirms the feasibility and functionality of the glass-slide FEIA platform for IL-6 detection in complex biological matrices. However, when compared with the plate-based plasmonic setup, the glass-slide configuration shows relatively lower signal enhancement and sensitivity.

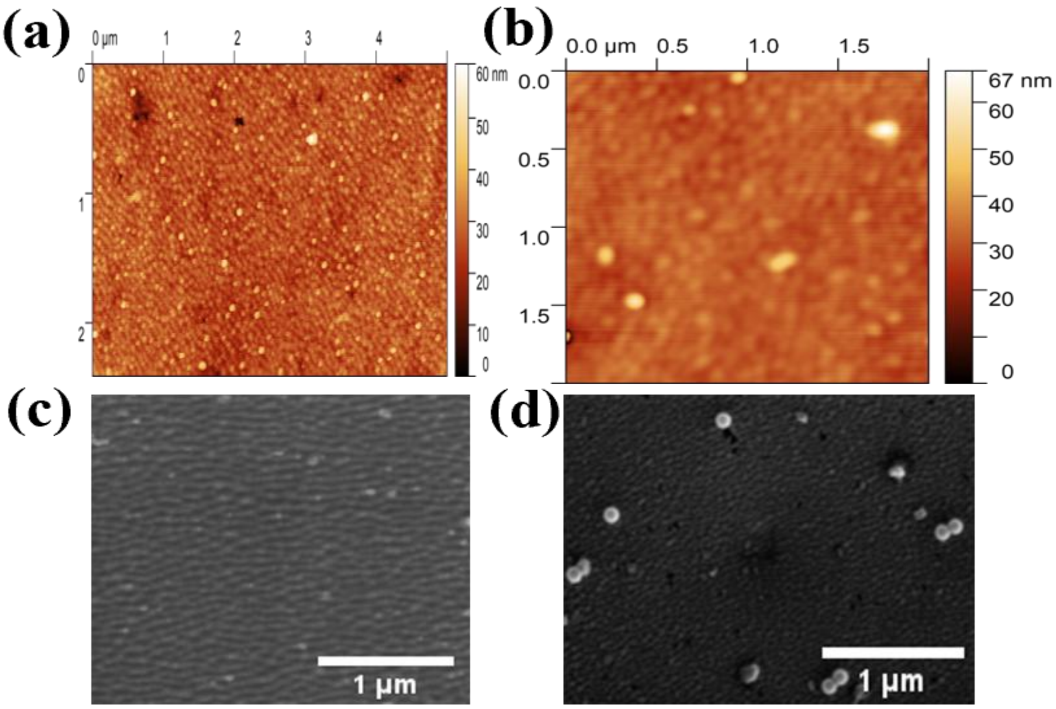

Figure S4. AFM and SEM images of functionalized plasmonic substrates before and after incorporation of AIE nanobeads in the immunoassay system: (a) AFM image of the plasmonic surface after immobilization of the capture antibody, (b) plasmonic surface after binding of streptavidin-labeled AIE nanobeads, (c) SEM image of the plasmonic surface after immobilization of the capture antibody, (d) plasmonic surface after binding of streptavidin-labeled AIE nanobeads

In figure S4 the AFM image of the antibody-functionalized plasmonic surface (panel a) shows a relatively uniform topography with small nanoscale features corresponding to the immobilized capture antibodies. After binding of streptavidin-labelled AIE nanobeads, the AFM image (panel b) displays a noticeable increase in surface roughness and distinct bright particulate spots, confirming the successful attachment of the nanobeads to the functionalized surface. Consistent with the AFM results, the SEM image of the antibody-coated plasmonic substrate (panel c) reveals a smooth nanostructured surface with no large aggregates. In contrast, after AIE nanobead binding (panel d), the surface shows clearly visible spherical particles distributed across the substrate, indicating effective incorporation of the fluorescent AIE nanobeads into the immunoassay system. These AFM and SEM results collectively verify each functionalization step and confirm that the plasmonic substrate efficiently captures the AIE nanobeads during immunoassay formation.

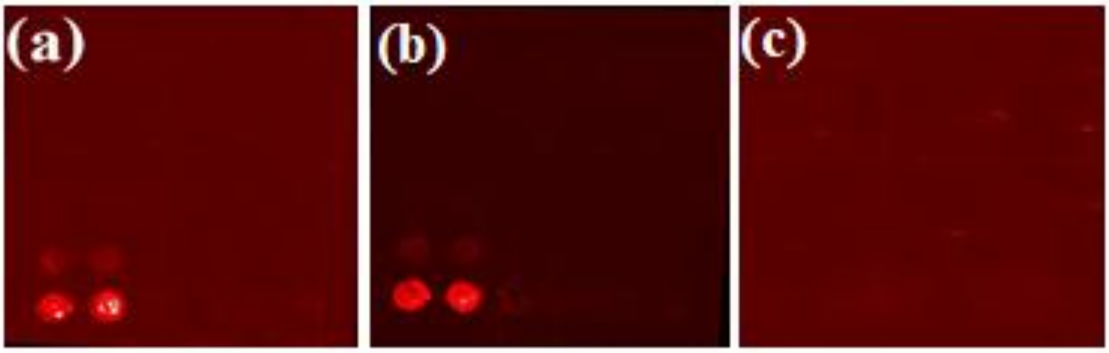

Figure S5: Comparison of nanobead performance on the developed plasmonic nanochip (a), a commercial substrate(b) and untreated PET substrate(c).

The figure S5 shows the fluorescent performance of AIE beads on our home made plasmonic substrate(a), a commercial plasmonic substrate(b) and untreated PET control(c). The images were captured using a Typhoon imager. The samples have been prepared by drop dry methods under the same concentration of three substrates. Each fluorescence spot corresponds to a specific concentration, arranged from the highest concentration at the bottom to zero concentration at the top. The intensity gradually decreases with decreasing particle concentration, demonstrating the expected dose-dependent response of the assay.

In comparison of image (a) and (b), the fluorescence signal from commercial substrate is visible but relatively weaker and less defined than that from the plasmonic substrate developed in this work. There is almost no signal found on the untreated PET substrate. The plasmonic nanochip produces significantly stronger fluorescence intensity, sharper spot definition, and an improved dynamic range. This enhanced performance highlights the advantage of the engineered plasmonic layer in amplifying fluorescence signals and enabling more sensitive detection.

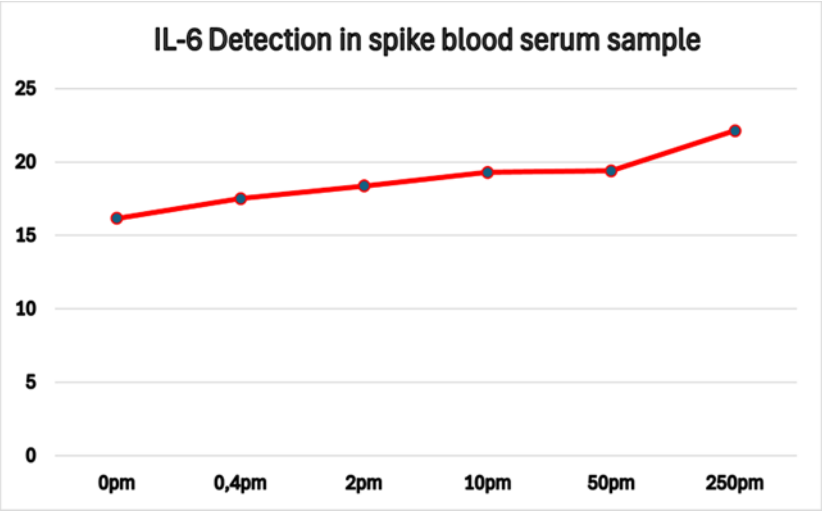

Figure S6: FEIA performance in spiked blood serum on glass slide setup.

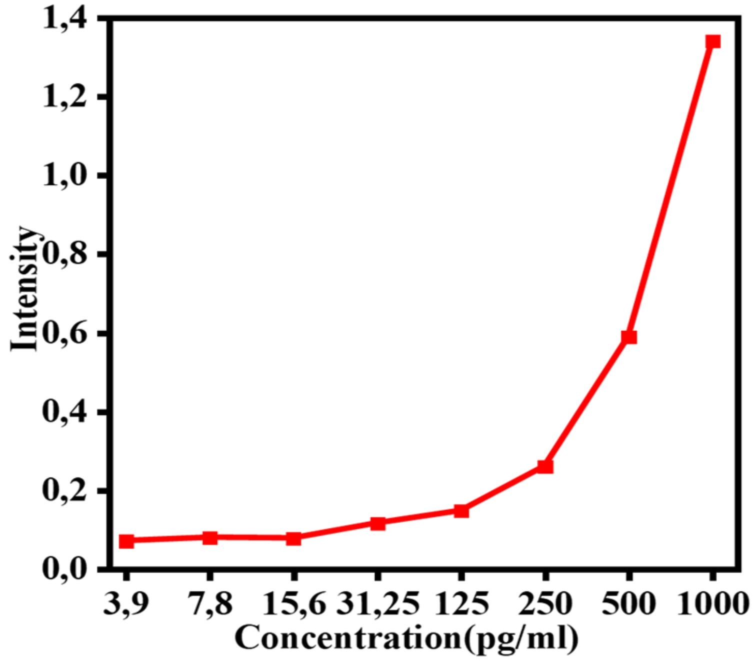

Figure S7: ELISA calibration and detection results for IL-6 using a commercial assay kit.
